# Quercetin Attenuates Atherosclerosis *via* Modulating Oxidized LDL-Induced Endothelial Cellular Senescence

**DOI:** 10.3389/fphar.2020.00512

**Published:** 2020-04-28

**Authors:** Yue-Hua Jiang, Ling-Yu Jiang, Yong-Cheng Wang, Du-Fang Ma, Xiao Li

**Affiliations:** ^1^Central Laboratory, Affiliated Hospital of Shandong University of Traditional Chinese Medicine, Jinan, China; ^2^First Clinical Medical College, Shandong University of Traditional Chinese Medicine, Jinan, China; ^3^Cardiovascular Department, Affiliated Hospital of Shandong University of Traditional Chinese Medicine, Jinan, China

**Keywords:** quercetin, endothelial cellular senescence, atherosclerosis, oxidized low-density lipoprotein, ApoE^-/-^ mice

## Abstract

**Background and Aims:**

Endothelial senescence is an important risk factor leading to atherosclerosis. The mechanism of quercetin against endothelial senescence is worth exploring.

**Methods:**

Quercetin (20 mg/kg/d) was administered to ApoE^-/-^ mice intragastrically to evaluate the effectiveness of quercetin on atherosclerotic lesion *in vivo*. *In vitro*, human aortic endothelial cells (HAECs) were used to assess the effect of quercetin on cellular senescence induced by oxidized low-density lipoprotein (ox-LDL). Transcriptome microarray and quantitative RT-PCR was conducted to study the pharmacological targets of quercetin.

**Results:**

ApoE^-/-^ mice demonstrated obvious lipid deposition in arterial lumina, high level of serum sIcam-1 and IL-6, and high density of Vcam-1 and lower density of Sirt1 in aorta. Quercetin administration decreased lipid deposition in arterial lumina, serum sIcam-1, and IL-6 and Vcam-1 in aorta, while increased the density of Sirt1 in aorta of ApoE^-/-^ mice. *In vitro*, quercetin (0.3, 1, or 3 μmol/L) decreased the expression of senescence-associated β-galactosidase and improved cell morphology of HAECs. And quercetin decreased the cellular apoptosis and increased mitochondrial membrane potential (ΔΨm) in dose-dependent manner, and decreased ROS generation simultaneously. Transcriptome microarray suggested 254 differentially expressed (DE) mRNAs (110 mRNAs were upregulated and 144 mRNAs were downregulated) in HAECs after quercetin treatment (fold change > 1.5, *P* < 0 .05, Que *vs* Ox-LDL). GO and KEGG analysis indicated nitrogen metabolism, ECM-receptor interaction, complement, and coagulation cascades, p53 and mTOR signaling pathway were involved in the pharmacological mechanisms of quercetin against ox-LDL.

**Conclusions:**

Quercetin alleviated atherosclerotic lesion both *in vivo* and *in vitro*.

## Introduction

Aging is an independent cardiovascular risk factor associated to endothelial dysfunction and arterial stiffness, which is a considered as very early event leading to cardiovascular disease (El Assar et al., 2012). Atherosclerotic plaque demonstrates evidence of cellular senescence. Endothelial senescence often leads to the specialized cell function disorder, apoptosis, and inflammation contributing to age-associated diseases, including atherosclerosis. A variety of vascular pathological factors induce endothelial cellular senescence. Among them, the more striking is oxidized low-density lipoprotein (ox-LDL) which induces oxidative stress and reduces telomerase activity in endothelial cells. The mitochondrial DNA (MtDNA) is susceptible to damage partly owing to the absence of protective histones within the DNA duplex, but also as a result of the close proximity to the ROS-producing inner mitochondrial membrane. Thus, both nuclear and mitochondrial DNA damage, defects in protein processing, intercellular communication dysfunction, and senescence -associated secretory phenotype are involved in cellular senescence (López-Otín et al., 2013; Uryga and Bennett, 2016).

Quercetin is a natural polyhydroxy flavonoid with wide distribution which has been recognized as a potential agent against diabetes, hypertension, cancer and neurodegenerative diseases. It has been also reported prominent effects of quercetin in hypertension, ischemia-reperfusion injury, and vascular senescence (Roos et al., 2016). The known pharmacological mechanisms of quercetin involves direct antioxidant effect, inhibiting endothelial-leukocyte adhesion, activation of Nrf2-ARE pathway (Costa et al., 2016; Wang et al., 2017), and attenuating the TLR-NF-κB signaling pathway (Bhaskar et al., 2016). However, the effectiveness and exact mechanism about quercetin against endothelial senescence in atherosclerosis remains unclear.

Herein, we focused on the effects of quercetin against atherosclerosis both *in vivo* and *in vitro*, and provide a global mRNA profile of in HAECs senescence treated with quercetin against ox-LDL by transcriptome microarray.

## Methods

### Animals

Fourteen specific pathogen free (SPF) grade male apolipoprotein E-deficient (ApoE^-/-^) mice and seven age-matched wild-type C57BL/6J (C57) mice of 8-week-old, weighing 18–22 g, were provided by Beijing Vital River Laboratory Animal Technology Co., Ltd (certificate: SCXC (Jing) 2016 - 0006). All mice were housed in an air-conditioned room with a 12 h light/dark cycle at a temperature of 21 ± 1°C and humidity of 50 ± 5% and got access to the diet and water *ad libitum*. The study was approved by Faculty of Animal Ethics Committee of Affiliated Hospital of Shandong University of Traditional Chinese Medicine (Jinan, China).

ApoE^-/-^ mice were fed a high-fat diet (Vital River, D12108c, Beijing, China) for 12 weeks to establish the atherosclerosis model, while C57 mice were fed a standard chow diet as control. Thenceforth, randomized seven of ApoE^-/-^ mice were administered with 20 mg/kg/d quercetin (Jiashi Yuhe Institute, 117-39-5, Beijing, China) intragastrically for 8 weeks; and the other seven ApoE^-/-^ mice and C57 mice were intragastrically given the same volume of saline. Mice were continued their same diet during intragastrically administration. Mice were sacrificed at the end of the 20^th^ week. After anesthesia with sodium pentobarbital (40 mg/kg, *i.p.*), blood was drawn by venipuncture and serum was separated centrifugally, and the aortas were removed on ice as soon as possible. The aortas were subjected to Oil red O staining and immunohistochemical staining.

### Oil Red O Staining

Atherosclerotic lesion and lipid deposition in arterial lumina was assessed using Oil red O staining. Briefly, the intact aortas were opened longitudinally to expose the intimal surface, fixed in 4% paraformaldehyde overnight, stained with 0.5% oil red O solution (Servicebio, G1015, Wuhan, China) for 1 hour, and then differentiated in a 60% propylene glycol solution for 5 min. The stained aortas were photographed on a black background using a digital camera under identical light conditions and photographing parameters.

### Enzyme-Linked Immunosorbent Assay (ELISA)

The level of serum soluble intercellular adhesion molecule-1 (sIcam-1) (Abcam, ab100688, Hong Kong, China) and interleukin-6 (IL-6) (Abcam, ab100712, Hong Kong, China) was determined by ELISA kits. All assays were performed following the manufacturer's instructions. Absorption values were detected at 450 nm. All samples were assayed in triplicate.

### Immunohistochemical Staining (IHC)

The density of vascular cell adhesion molecule-1 (Vcam-1) and sirtuin 1 (Sirt1) in aorta was determined by IHC. Aortas were paraffin-embedded and tissue sections (4 μm thick) were immunohistochemical stained at room temperature following standard procedures. Briefly, after 3% H_2_O_2_ incubating for elimination of endogenous peroxidases, microwaving for antigen retrieval and 5% BSA blocking, sections were incubated with rabbit anti-mouse Vcam-1 primary antibody (1:800 dilution; Proteintech, 11444-1-AP, Wuhan, China) or rabbit anti-mouse Sirt1 primary antibody (1:100 dilution; Proteintech, 13161-1-AP, Wuhan, China) in a moisture box at 4°C overnight. And then incubated with goat anti-rabbit IgG secondary antibodies (1:500 dilution; Proteintech, SA00004-2, Wuhan, China) at room temperature for 60 min at the next morning. Color was developed using DAB and counterstained with hematoxylin. Each section was observed under a light microscope and the sites with brown-yellow granule precipitation were judged as positive.

### Cell Culture

Human aortic endothelial cells (HAECs, ScienCell, 6100, USA) were cultured in a endothelial cell medium (ECM, ScienCell, 1001, USA) supplemented with 1% endothelial cell growth supplement (ECGS), 5% fetal bovine serum (FBS), and 1% penicillin/streptomycin (P/S) on culture dishes coated with human fibronectin at 37°C in a humidified 5% CO_2_ incubator. HAECs were cultured for 48 h in the presence of ox-LDL (50 μg/mL, Xiesheng Biotechnology, Beijing, China), or ox-LDL (50 μg/mL) + quercetin at concentrations of 3, 1, or 0.3 μmol/L, and untreated HAECs was used as normal control. All samples were assayed in triplicate under safety and sterile processes during the cellular experiments.

### β-Galactosidase (SA-β-G) Staining

Elevated expression of senescence-associated β-galactosidase is one of multiple features of senescence (Wang et al., 2015). Cellular senescence was assayed by the Senescence SA-β-G Staining Kit (Beyotime, C0602, Shanghai, China) following the manufacturer's instructions. Briefly, HAECs were fixed with 1 ml fixation buffer for 15 min at room temperature. After washing, cells were incubated with 1 ml staining mixture overnight in 37°C. Light microscopic pictures were acquired under microscope (Zeiss, Axio Vert.A1, Germany) and quantified by Image J software. The cells dyed blue were considered senescent cells. Senescent rate (%) = number of senescent cells/total cells × 100.

### Transmission Electron Microscopy (TEM)

The HAECs were collected, fixed in 2.5% glutaraldehyde solution and then observed under TEM to evaluate the ultrastructural changes. Sample processing and image capture were performed by Weiya Biotechnology Co., Ltd (Jinan, China).

### Mitochondrial Labeling

Mito-Tracker Green (Beyotime, Haimen, China), a selective lipophilic dye, was used to label active mitochondria. HAECs were incubated with 150 nmol/l Mito-Tracker Green for 30 min, and then observed by fluorescence microscopy (490 nm of excitation wavelength, 516 nm of emission wavelength, Zeiss, Axio Vert.A1, Germany).

### MTT Assay

MTT assay was performed to determine cellular viability. MTT (5 mg/ml) was added to 96-well plate during the last 4 h of incubation, medium was replaced with 150 µl DMSO to dissolve the formazan crystals and the absorbance was measured at 562 nm with a reference wavelength of 630 nm.

### Cell Apoptosis Assay

Cellular apoptosis rate was evaluated with the Annexin V-FITC/PI Apoptosis Detection Kit (EMD Millipore, MCH100105, Billerica MA, USA) following the manufacturer's protocol and analyzed by flow cytometry (Merck Millipore, Muse, Darmstadt, Germany).

### Intracellular Reactive Oxygen Species (ROS) Generation

Intracellular ROS level was determined by a Reactive Oxygen Species Assay Kit (Beyotime, S0033, Shanghai, China). HAECs in 6-well plates were loaded with 10 μmol/L 2',7'-dichlorofluorescin diacetate (DCFH-DA), at 37°C for 25 min. After washing steps, observed under Zeiss Vert A1 fluorescence microscope and measured using a flow cytometer (BD Accuri C6, USA) with excitation at 488 nm and emission at 525 nm. Relative ROS generation (%) = fluorescence treatment/fluorescence control × 100.

### Detection of Mitochondrial Membrane Potential (ΔΨm)

Mitochondrial membrane disruption and potential was assayed by Mitochondrial Membrane Potential Assay Kit with JC-1 following the manufacturer's instructions (Beyotime, C2006, Shanghai, China). In brief, HAECs were trypsined and collected, incubated with JC-1 staining mixture at 37°C for 20 min, and subjected to flow cytometer (BD Accuri C6, USA) with excitation at 485 nm and emission at 590 nm (Song et al., 2017).

### Transcriptome Microarray

A mRNA profile in HAECs senescence treated with quercetin at optimum concentration (3 μmol/L) against ox-LDL was assayed by transcriptome microarray. Total RNA of HAECs was extracted by the TRIzol Reagent Kit (Life Technologies, 15596-018, Carlsbad, USA) following the manufacturer's instructions. RNA was purified by RNeasy mini kit (QIAGEN, 74106, GmBH, Germany) and RNase-Free DNase Set (QIAGEN, 79254, GmBH, Germany). RNA was amplified and labeled using Low Input Quick Amp WT Labeling Kit (Agilent, 5190-2943, Santa Clara, CA, US). 1.65μg Cy3-labeled cRNA on slide was hybridized by Gene Expression Hybridization Kit (Agilent, 5188-5242, Santa Clara, CA, US) in a hybridization oven for 17 h hybridization. Slides were scanned by Agilent Microarray Scanner and data were obtained with Feature Extraction software 10.7 (Agilent technologies, Santa Clara, CA, US). Raw data were normalized by Quantile algorithm, limma packages in R. Transcriptome microarray assays were performed by the Biotechnology Corporation (Shanghai, China). Differentially expressed (DE) genes were analyzed with the DESeq2 R package (1.10.1). Gene Ontology (GO) and Kyoto encyclopedia of genes and genomes (KEGG) pathway analysis were performed by clusterProfiler R package to test the statistical enrichment of DE genes.

### Quantitative Reverse Transcription Polymerase Chain Reaction (qRT-PCR)

Six key DE genes were selected for qRT-PCR, including AATK, CDKN2A, DPY19L4, EIF4E1B, IGFBP3, MPP5, and SLC5A11. The sequences for primers are listed in **Table 1**. Total RNA was isolated and qRT-PCR was performed using standard protocols. In detail, total RNA was extracted using TRIzol reagent (Invitrogen, Carlsbad, CA, USA) and synthesized into cDNA from 2 ng of total RNA using the PrimScript RT reagent Kit (Roche, Basel, Switzerland). The relative mRNA expression level was normalized to β-actin. All data were analyzed by 2^-ΔΔCT^ method. All qRT-PCR were assayed in a LightCyler 480 system (Roche, Basel, Switzerland).

**Table 1:** The sequences for primers for q-PCR.

Gene Symbol	Forward primer (5'-3')	Reverse primer (5'-3')
AATK	ACCTTCATCGCAACAATTTCGT	AGTAGTCCTCTCTGTACTTGCAG
CDKN2A	GATCCAGGTGGGTAGAAGGTC	CCCCTGCAAACTTCGTCCT
DPY19L4	ACCATGAACGGAAATTCTGGTT	TGTGTCAGTTCGTAAACACCTCT
EIF4E1B	GACAAGATCGCTGTGTGGACGA	GTTGCTCTTGGTGGCTGTGTCT
IGFBP3	CGCTACAAAGTTGACTACGAGTC	GTCTTCCATTTCTCTACGGCAGG
MPP5	ATGATGCCAATACGTCGAAGTG	CTCTCTCCTACGCCTCATGTC
SLC5A11	CCAAACTCGTGCTGGAACTCCT	GTGAAGATGGTGCTGGCACTGT
β-actin	CACCATTGGCAATGAGCGGTTC	AGGTCTTTGCGGATGTCCACGT

### Western Blotting Analysis

For validation of the data of transcriptome microarray and qRT-PCR, EIF4E1B, IGFBP3, and SLC5A11 were chosen for Western blot assay. HAECs were collected and lysed in RIPA lysis buffer (Beyotime, P0013, Nantong, China). 40 μg protein samples were subjected to 12% sodium dodecyl sulfate polyacrylamide gel electrophoresis, transferred onto PVDF membranes, and then blocked with 5% nonfat dry milk in PBS-0.05% Tween-20 (PBS-T) for 1 h at room temperature. The blots were incubated at 4°C with gentle shaking overnight with primary antibodies (Bioss, Rabbit Anti-EIF4E1B antibody, bs-4979R, 1:1000, Beijing, China; Proteintech, Rabbit Anti-IGFBP3 antibody, 10189-2-AP, 1:500, Wuhan, China; Proteintech, Rabbit Anti-IGFBP3 antibody, 14089-1-AP, 1:500, Wuhan, China). After washing steps, the blots were incubated with horseradish peroxidase conjugated to goat anti-rabbit IgG (1:20,000) at room temperature for 1 h. They were then visualized by incubation with Immobilon Western Chemiluminescent HRP Substrate (Merck Millipore, 1579205, Darmstadt, Germany) and exposed for 60 s using Fluor Chem Q system. The bands were quantifyed by Image J (National Institutes of Health, Bethesda, Maryland, USA) and the density value was normalized to that of β-actin.

### Statistical Analysis

Statistical analysis was performed by using SPSS 22.0. The data were collected and shown as mean ± standard deviation (SD). Statistical analysis was performed by one-way ANOVA, followed by LSD test. *P* < 0.05 was considered to be statistically different.

## Results

### Quercetin Alleviated Atherosclerotic Lesion *In Vivo*

**Figure 1:**
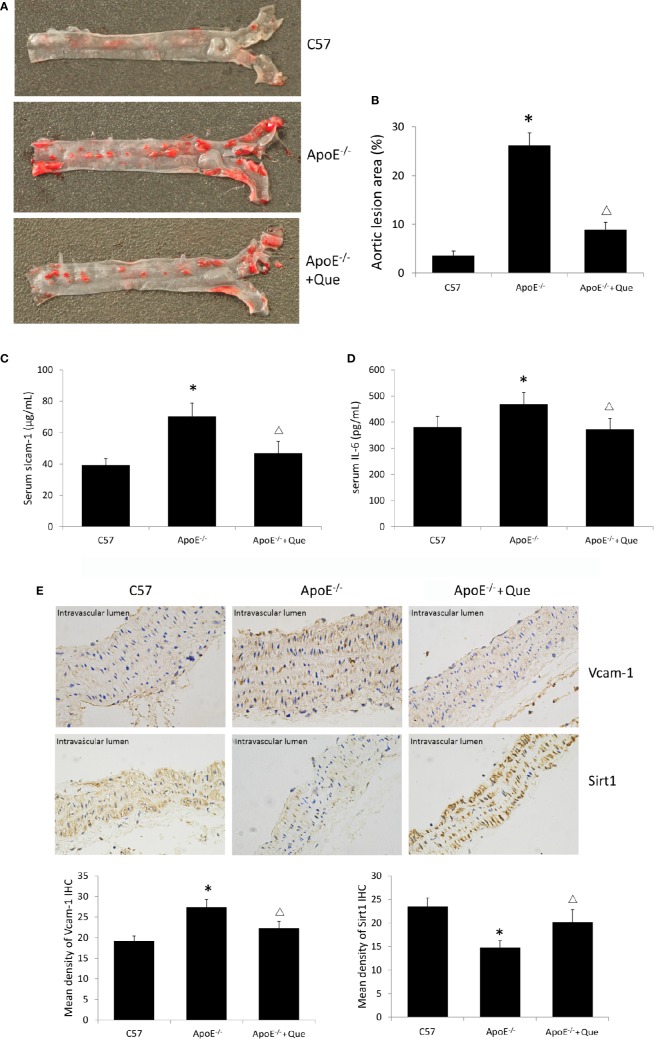
Quercetin alleviated atherosclerotic lesion *in vivo*. **(A)** Oil red O staining of entire arterial tree to assess lipid deposition and atherosclerotic lesion of mice (n = 3). **(B)** Quantification of the gross lesion areas in the entire arterial tree (n = 3). **(C)** Serum soluble Icam-1 level was determined by enzyme-linked immunosorbent assay (ELISA) assay (n = 7). **(D)** Serum IL-6 level was determined by ELISA assay (n = 7). **(E)** the representative photograph and semi-quantitative analysis of IHC of density of Vcam-1 and Sirt1 in aorta (n = 3). The sites in brown-yellow colour were judged as positive (× 400). *P < 0.05, *vs* C57 mice; ^Δ^*P* < 0.05, *vs* ApoE^-/-^ mice.

Oil red O staining demonstrated obvious lipid deposition in arterial lumina and severe atherosclerotic lesion in ApoE^-/-^ mice, while 8-week-quercetin-administration decreased Oil red O positive scope (**Figures 1A, B**). And quercetin significantly decreased the level of serum sIcam-1 and IL-6 in ApoE^-/-^ mice (*P* < 0.05, **Figures 1C, D**). The photograph and semi-quantitative analysis of IHC sugggested that ApoE^-/-^ mice had higher density of Vcam-1 and lower density of Sirt1 in aorta than that of C57, while quercetin decreased the density of Vcam-1 and increased the density of Sirt1 effectively (*P* < 0.05, **Figure 1E**).

### Quercetin Alleviated HAECs Senescence

**Figure 2:**
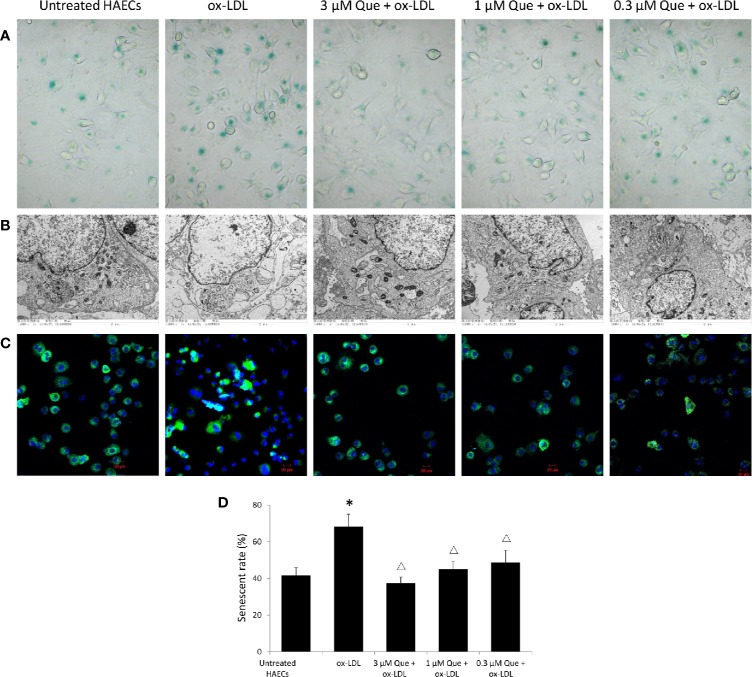
Quercetin alleviated human aortic endothelial cells (HAECs) senescence *in vitro*. HAECs were cultured for 48 h in the presence of 50 μg/mL ox-LDL, or 50 μg/mL ox-LDL followed with different quercetin (3, 1 or 0.3 μmol/L), and untreated HAECs was used as normal control. **(A)** HAECs senescence was assessed by SA-β-G staining. **(B)** Subcellular morphology was observed under transmission electron microscopy (TEM). **(C)** Fluorescence images of mitochondria by Mito Tracker Green as molecular probe. **(D)** HAECs senescence rate was semi-quantitative analyzed according to SA-β-G staining. *P < 0.05, *vs* untreated HAECs; ^Δ^*P* < 0.05, *vs* ox-LDL; n = 3.

*In vitro*, SA-β-G staining suggested that ox-LDL increased HAECs senescence rate by 63.5%, while 3 μmol/L quercetin effectively decreased senescence rate by 45.2% (*P* < 0.05, **Figures 2A, D**). The untreated HAECs showed normal morphology, uniform chromatin distribution in the nucleus and abundant mitochondria, and well developed endoplasmic reticulum. The senescent HAECs induced by ox-LDL was shrinked, the chromatin was concentrated, the number of mitochondria was decreased, enlargement of the lysosomal compartment and focal demyelination was observed. After the quercetin intervention, the deposition of lipid droplets was decreased, lysosomal compartment was normalized and the cell morphology was improved (**Figure 2B**). The distribution of mitochondria in normal cells was homogeneitic and abundant. Some of the mitochondria was concentrated and some disappeared after ox-LDL induction. Quercetin administration, especially 3 and 1 μmol/L quercetin, recovered the morphology of mitochondria (**Figure 2C**).

### Effect of Quercetin on Apoptosis, ROS, and ΔΨm

**Figure 3:**
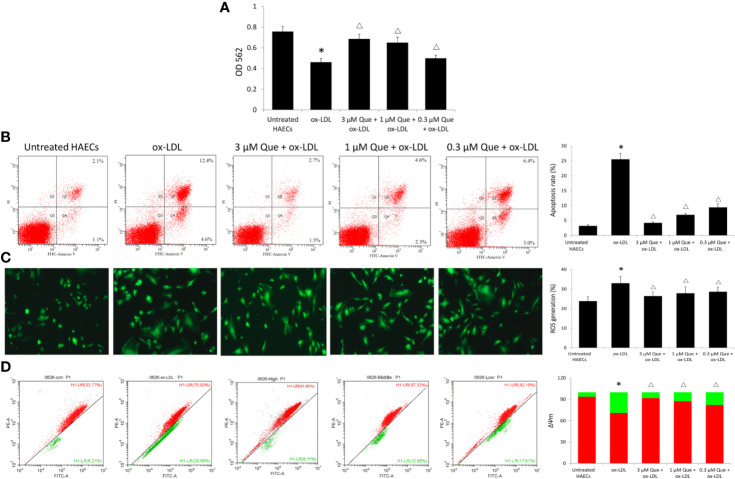
Effect of Quercetin on apoptosis, reactive oxygen species (ROS), and ΔΨm. Human aortic endothelial cells (HAECs) were cultured for 48 h in the presence of 50 μg/ml ox-LDL, or 50 μg/ml ox-LDL followed with different quercetin (3, 1 or 0.3 μmol/L), and untreated HAECs was used as normal control. **(A)** viability of HAECs was determined by MTT assay. **(B)** Apoptosis rate was determined by Annexin V-FITC/PI. **(C)** ROS generation was determined by 2',7'-dichlorofluorescin diacetate (DCFH-DA). **(D)** The degree of mitochondrial depolarization and ΔΨm was assayed by JC-1 staining and ΔΨm was assessed by the relative ratio of red fluorescence to green fluorescence *via* flow cytometer. ΔΨm reversibly changes color from green to red as the membrane potential increases (values of > 80–100 mV). *P < 0.05, *vs* untreated HAECs; ^Δ^*P* < 0.05, *vs* ox-LDL; n = 3.

Ox-LDL decreased the viability of HAECs by 64.6%, while 3, 1, or 0.3 μmol/L quercetin increased the viability of HAECs by 48.9%, 40.9%, and 10.8%, respectively (**Figure 3A**). Untreated HAECs had an apoptosis rate of 3.18%, the apoptosis rate was increased 8 times to 25.5% as ox-LDL exposure, especially early apoptosis rate, while quercetin decreased the cellular apoptosis in dose-dependent manner (*P* < 0.05, **Figure 3B**). Untreated HAECs demonstrated 23.86% ROS generation, ox-LDL increased ROS generation to 32.96, while quercetin at different concentrations showed a similar reduction in ROS generation (*P* < 0.05, **Figure 3C**). The degree of mitochondrial depolarization and ΔΨm is ususally judged by the relative ratio of red fluorescence to green fluorescence *via* flow cytometer. Ox-LDL exposure decreased ΔΨm significantly, whlie quercetin increased the ΔΨm in dose-dependent manner (*P* < 0.05, **Figure 3D**).

### MRNA Profile After Quercetin Treatment and qRT-PCR and Western Blotting Verification

**Figure 4:**
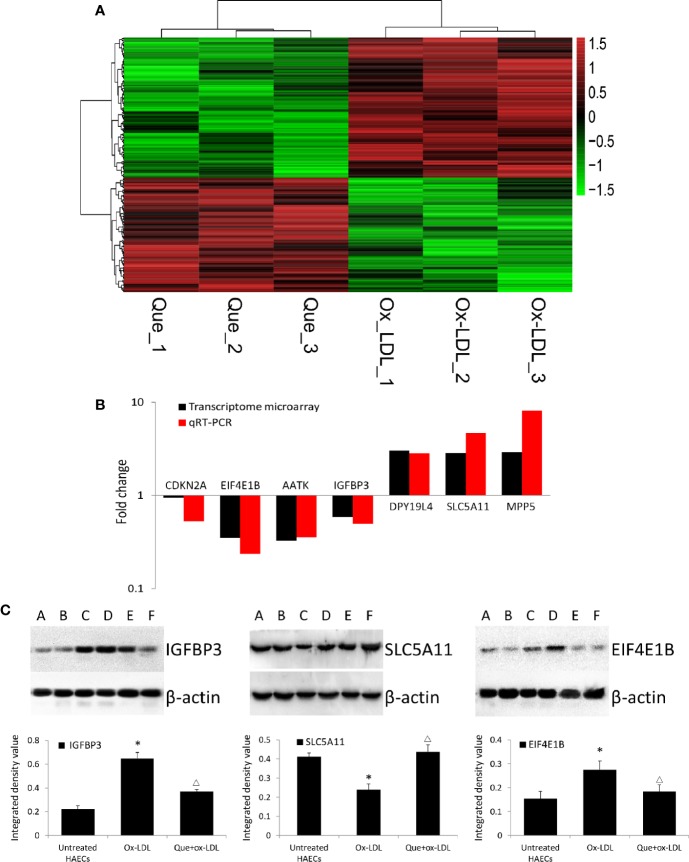
Heatmap of differentially expressed (DE) mRNAs in transcriptome microarray and q-PCR and Western blotting verification. **(A)** Heatmap of DE mRNAs in transcriptome microarray (n = 3). Human aortic endothelial cells (HAECs) were cultured for 48 h in the presence of 50 μg/mL ox-LDL, or 50 μg/mL ox-LDL followed with quercetin at optimum concentration (3 μmol/L). In total, 254 DE mRNAs (110 mRNAs were upregulated and 144 mRNAs were downregulated) were identified (fold change > 1.5, *P* < 0 .05, Que *vs* Ox-LDL). Black stands for 0, meaning no difference between groups. Red stands for high expression, while green represents low expression. **(B)** q-PCR (n = 6) verification DE mRNAs compared to microarrays. **(C)** Western blotting assay and integrated density analysis verified the DE protein expression level of EIF4E1B, IGFBP3 and SLC5A11. A and B: Untreated HAECs; C and D: Ox-LDL treated HAECs; E and F; ox-LDL treatment followed with 3 mmol/L quercetin. *P < 0.05, vs untreated HAECs; ΔP < 0.05, vs ox-LDL; n = 6.

In total, 254 DE mRNAs (among them, 110 mRNAs were upregulated and 144 mRNAs were downregulated) were identified in HAECs after Ox-LDL exposure followed by 3 μmol/L quercetin treatment (fold change > 1.5, *P* < 0 .05, Que *vs* Ox-LDL). The DE mRNAs are shown in a heatmap (**Figure 4A**). The top 10 up- or down-regulated mRNAs are listed in **Table 2**. The mRNA expression of CDKN2A, EIF4E1B, AATK, and IGFBP3 was decreased, while that of DPY19L4, SLC5A11, and MPP5 was increased in qRT-PCR, which were consistent with the microarrays results (**Figure 4B**). Among them, the protein expression of EIF4E1B and IGFBP3 was decreased, while that of SLC5A11 increased after quercetin administration (**Figure 4C**).

**Table 2:** The top 10 up-/down-regulated mRNAs in transcriptome microarrays (n = 3, Que vs Ox-LDL).

Gene Symbol	*P* values	Fold change	Regulation	Description
DPY19L4	0.0302	3.0306	Up	dpy-19-like 4 (C. elegans)
MPP5	0.0286	2.9217	up	membrane protein, palmitoylated 5 (MAGUK p55 subfamily member 5), transcript variant 1
SLC5A11	0.0136	2.8538	up	solute carrier family 5 (sodium/inositol cotransporter), member 11, transcript variant 2
CIITA	0.0069	2.8402	up	class II, major histocompatibility complex, transactivator, transcript variant 1
HMP19	0.0118	2.8340	up	HMP19 protein
SLC6A12	0.0146	2.6643	up	solute carrier family 6 (neurotransmitter transporter), member 12, transcript variant 1
ADCYAP1R1	0.0159	2.6621	up	adenylate cyclase activating polypeptide 1 (pituitary) receptor type I, transcript variant 1
GOLGA6L2	0.0000	2.5747	up	golgin A6 family-like 2
RAB39B	0.0224	2.5377	up	RAB39B, member RAS oncogene family
NPC1L1	0.0311	2.4792	up	NPC1-like 1, transcript variant 1
TPTE	0.0052	0.4015	down	transmembrane phosphatase with tensin homology, transcript variant 1
BAALC	0.0017	0.3921	down	brain and acute leukemia, cytoplasmic, transcript variant 1
LELP1	0.0238	0.3850	down	late cornified envelope-like proline-rich 1
NMU	0.0057	0.3644	down	neuromedin U, transcript variant 1
EIF4E1B	0.0076	0.3498	down	eukaryotic translation initiation factor 4E family member 1B
ONECUT3	0.0408	0.3340	down	one cut homeobox 3
AATK	0.0216	0.3278	down	apoptosis-associated tyrosine kinase, transcript variant 1
SPATS2	0.0054	0.2652	down	spermatogenesis associated, serine-rich 2, transcript variant 1
PLGLB1	0.0260	0.2557	down	plasminogen-like B1
SEZ6	0.0049	0.1380	down	seizure related 6 homolog (mouse), transcript variant 1

**Figure 5:**
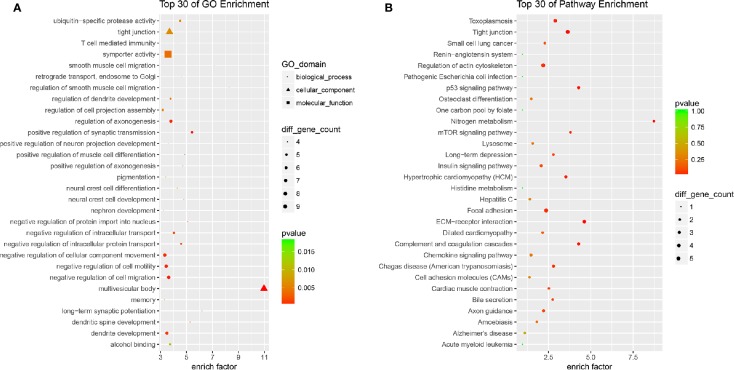
Bioinformatics analysis of differentially expressed (DE) mRNAs. **(A)** Top 30 of Gene Ontology (GO) enrichment of quercetin *vs* ox-LDL. **(B)** Top 30 of KEGG enrichment of quercetin *vs* ox-LDL.

All the 254 DE mRNAs were subjected for bioinformatic analysis. In bioinformatics analysis, gene product annotations are divided into three categories: biological processes (BP), cellular components (CC), and molecular functions (MF) in GO enrichment. In the present study, GO analysis indicated multivesicular body (GO ID: GO: 0005771; Type: CC; P value: 2.692e-05), regulation of smooth muscle cell migration (GO ID: GO: 0014910; Type: BP; P value: 3.906e-04), long-term synaptic potentiation (GO ID: GO: 0060291; Type: BP; P value: 1.322e-03), positive regulation of synaptic transmission (GO ID: GO: 0050806; Type: BP; P value: 3.213e-04), and dendritic spine development (GO ID: GO: 0060996; Type: BP; P value: 2.578e-03) contributed to efficacy of quercetin (**Figure 5A**). KEGG analysis suggested nitrogen metabolism, ECM-receptor interaction, complement and coagulation cascades, p53 signaling pathway, and mTOR signaling pathway were involved in the pharmacology of quercetin against ox-LDL (**Figure 5B**).

## Discussion

Endothelial cells usually work as the first defensive line in vessels, which are currently considered as a central role in cardiovascular homeostasis. Although cell senescence demonstrates a definite tumor suppression effect (Muñoz-Espín and Serrano, 2014), long-term accumulation of senescent cells may impede the regeneration and maintenance of renewable tissues (Ogrodnik et al., 2019). Generally known, senescence of endothelial cells increases the risk of various cardiovascular diseases including atherosclerosis. Endothelial senescence always leads to endothelial dysfunction which is regarded as an initial event in the development of atherosclerotic plaques. It has been verified the presence of vascular endothelial cells with senescence-associated phenotypes in the atherosclerotic regions of human coronary arteries (Minamino et al., 2002). Therefore, therapies to reverse the endothelial aging process may function as an important strategy for the treatment of atherosclerosis.

Quercetin, widely present in fruits and vegetables as a secondary plant metabolite, is charactered by antidiabetic, vascular protection, neuroprotection, and anticarcinogenic potential. Quercetin regulates blood pressure and preserves cardiovascular function *via* varying vascular compliance, peripheral vascular resistance, total blood volume, anti-inflammatory, and anti-oxidant actions (Terao, 2017). On the basis of the structure and property of the molecules, quercetin is beneficial to the circulatory system, vessels, and the blood flow (Lichota et al., 2019). In recent years, the effect of quercetin has attracted more attention. Jia et al. reported that quercetin treatment significantly reduced the atherosclerotic plaque area and lipid accumulation in aorta, together with decreased serum levels of LDL-C, ox-LDL, TNF-α, and IL-6 in ApoE^-/-^ mice (Jia et al., 2019). Lu et al. reported that quercetin inhibited atherosclerotic plaque development in C57BL/6 mice by inhibiting ROS production and activating the PI3K/AKT signaling pathway (Lu et al., 2017). Furthermore, Cao et al. reported quercetin treatment ameliorated substantial atherosclerosis pathology, induced autophagosomes, decreased the level of TNF-α, IL-1β, IL-18 in ApoE^-/-^ mice (Cao et al., 2019). Our results confirmed again the anti-atherosclerosis effect of quercetin. In the present study, 20 mg/kg/d quercetin administration for 8 weeks effectively alleviated lipid deposition in arterial lumina and atherosclerotic lesions. Senescent cells are characterized by senescence-associated secretory phenotype (SASP), secreting pro-inflammatory cytokines, chemokines, proteases, and other factors. Thus, we assessed the effect of quercetin on adhesion molecules and inflammatory mediator which contributes to senescence-associated systemic dysfunction and atherosclerosis. SIRT1, a key molecule for basal level autophagy, declines during senescence progression and, this change of SIRT1 is responsible for the similar decline of mitophagy during senescence (Sasaki et al., 2006; Song et al., 2017). Both local and circulating adhesion molecules and inflammatory factors contribute to vascular aging and atherosclerosis. Our data supported that quercetin significantly decreased the level of serum sIcam-1 and IL-6, and Vcam-1 in aorta, while increased the density of Sirt1 in aorta of ApoE^-/-^ mice.

Senescence is an irreversible growth arrest which is triggered by stresses such as oxidative reaction, telomere shortening or DNA damage. Oxidized LDL is regarded as a key factor of oxidative stress and endothelial dysfunction, which can directly induce endothelial senescence and endothelial dysfunction (Rodríguez-Mañas et al., 2009). Ox-LDL is candidate biomarkers in evaluating the human biological age (Gradinaru et al., 2015). It has been reported that quercetin prevented atherosclerosis by regulating lipid metabolism, lessening ox-LDL-induced lipid deposition in macrophages, promoting macrophage cholesterol efflux, and blocking foam cell formation. Increased β-galactosidase activity is regarded as one of markers of senescent cells. In the present study, quercetin decreased the expression of senescence-associated β-galactosidase and improved cell morphology of HAECs. And quercetin decreased the cellular apoptosis and increased ΔΨm in dose-dependent manner, and decreased ROS generation simultaneously. Our data confirmed that quercetin alleviated HAECs senescence *in vitro* effectively.

We tried to explore the potential mechanisms of quercetin against ox-LDL by transcriptome microarray and identified 254 DE mRNAs in HAECs after quercetin administration. Quercetin decreased the expression of AATK, CDKN2A, EIF4E1B, and IGFBP3. AATK is induced during apoptosis. CDKN2A (p16) is one of the most important senescence markers. EIF4E1B promotes RNA transport and initiation of protein synthesis. High circulating IGFBP-3 concentration predicts greater incidence of cardiovascular conditions including ischemic heart disease and congestive heart failure and high risk of cancer (Juul et al., 2002; Schumacher et al., 2010). These data supported that quercetin decreased apoptosis, regulated nitrogen balance, and reduced the risk of cardiovascular events. Quercetin increased the expression of DPY19L4, MPP5, and SLC5A11. DPY19L4 is localized in endoplasmic reticulum (ER) which involves in regulation of glycosylation of thrombospondin type 1 repeats (TSRs). MPP5 plays a role in GABA uptake by stabilizing the transporter, the establishment of cell polarity, and the dynamic remodeling of the apical cytoskeleton. SLC5A11 is exclusively responsible for apical myo-inositol transport and absorption in intestine. These data supported that quercetin improved function of ER and molecule transportation and maintained cytoskeleton. DE mRNAs indicated multivesicular body, regulation of smooth muscle cell migration, long-term synaptic potentiation, positive regulation of synaptic transmission, dendritic spine development contributed to efficacy of quercetin. KEGG analysis suggested nitrogen metabolism, ECM-receptor interaction, complement, and coagulation cascades, p53, and mTOR signaling pathway were involved in the pharmacology of quercetin against ox-LDL. Both p53 and mTOR signaling pathway are key mediators regulating aging. The establishing and maintaining the senescence growth arrest is known to be dependent on the p53 signaling pathway (Campisi and d'Adda, 2007). mTOR has been claimed to be key molecule in switching on/off senescence/quiescence and partial p53 activation sustains mTOR activity and causes senescence subsequently (Kiyono, 2007). Quercetin has been identified as senolytic drug against senescent human endothelial cells and mouse BM-MSCs (Zhu et al., 2015; Kirkland et al., 2017). Age-related loss of protein homeostasis is further increased as senescence occurrance. It is known that various amino acids, especially leucine, are the nutrient signals for mTOR. Huang et al. reported that quercetin effectively suppressed amino acid-induced mTORC1 activation and worked as a competitive mTOR kinase inhibitor at the ATP-binding motif in cultured retinal pigment epithelial (RPE) cells (Huang et al., 2018). In the present study, quercetin forcefully regulated both p53 and mTOR signaling pathway, as well as nitrogen metabolism. Thus, we concluded that the regulation of nitrogen metabolism contributed to the mediation of quercetin on mTOR pathway. Understanding and modulating these processes might provide novel strategy to influence senescence and aging-related diseases including atherosclerosis.

## Conclusions

Quercetin alleviated atherosclerotic lesion both *in vivo* and *in vitro*. Nitrogen metabolism, ECM-receptor interaction, complement and coagulation cascades, p53, and mTOR signaling pathway were involved in the mechanisms of quercetin against ox-LDL.

## Data Availability Statement

The data generated for this study can be found here: https://www.ncbi.nlm.nih.gov/geo/query/acc.cgi?acc=GSE139288.

## Ethics Statement

The animal study was reviewed and approved by Faculty of Animal Ethics Committee of Affiliated Hospital of Shandong University of Traditional Chinese Medicine.

## Author Contributions

Y-HJ and XL designed the experiments. Y-HJ performed both zoology and cytology experiments, and drafted the manuscript. L-YJ presided at cytology experiments. Y-CW performed zoology experiments and animal care. D-FM collected samples and analyzed data. XL revised the manuscript. All authors reviewed and approved the final manuscript.

## Funding

The present study was funded by National Natural Science Foundation of China No. 81673807 to Y-HJ and National Natural Science Foundation of China No. 81673970 to XL.

## Conflict of Interest

The authors declare that the research was conducted in the absence of any commercial or financial relationships that could be construed as a potential conflict of interest.
