# Corrigendum: Quercetin Attenuates Atherosclerosis *via* Modulating Oxidized LDL-Induced Endothelial Cellular Senescence

**DOI:** 10.3389/fphar.2020.00772

**Published:** 2020-05-29

**Authors:** Yue-Hua Jiang, Ling-Yu Jiang, Yong-Cheng Wang, Du-Fang Ma, Xiao Li

**Affiliations:** ^1^Central Laboratory, Affiliated Hospital of Shandong University of Traditional Chinese Medicine, Jinan, China; ^2^First Clinical Medical College, Shandong University of Traditional Chinese Medicine, Jinan, China; ^3^Cardiovascular Department, Affiliated Hospital of Shandong University of Traditional Chinese Medicine, Jinan, China

**Keywords:** quercetin, endothelial cellular senescence, atherosclerosis, oxidized low-density lipoprotein, ApoE^-/-^ mice

In the original article, there was a mistake in Figure 3 as published. The wrong image of ROS generation in the 3 μm Que+ox-LDL group was unintentionally used in **Figure 3C**. The fully corrected **Figure 3** appears below.

**Figure 3:**
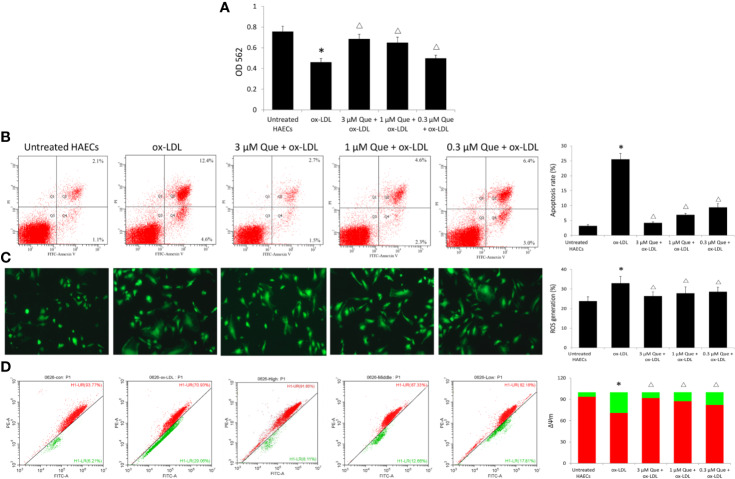
Effect of Quercetin on apoptosis, reactive oxygen species (ROS), and ΔΨm. Human aortic endothelial cells (HAECs) were cultured for 48 h in the presence of 50 μg/ml ox-LDL, or 50 μg/ml ox-LDL followed with different quercetin (3, 1 or 0.3 μmol/L), and untreated HAECs was used as normal control. **(A)** viability of HAECs was determined by MTT assay. **(B)** Apoptosis rate was determined by Annexin V-FITC/PI. **(C)** ROS generation was determined by 2',7'-dichlorofluorescin diacetate (DCFH-DA). **(D)** The degree of mitochondrial depolarization and ΔΨm was assayed by JC-1 staining and ΔΨm was assessed by the relative ratio of red fluorescence to green fluorescence *via* flow cytometer. ΔΨm reversibly changes color from green to red as the membrane potential increases (values of > 80–100 mV). *P < 0.05, *vs* untreated HAECs; ^Δ^*P* < 0.05, *vs* ox-LDL; n = 3.

The authors apologize for this error that this does not change the scientific conclusions of the article in any way. The original article has been updated.

